# Association of Meniscus Injuries in Patients With Anterior Cruciate Ligament Injuries

**DOI:** 10.7759/cureus.25878

**Published:** 2022-06-12

**Authors:** Sagar Venkataraman, Prabhu Ethiraj, Arun H Shanthappa, Kishore Vellingiri

**Affiliations:** 1 Department of Orthopaedics, Sri Devaraj Urs Medical College, Sri Devaraj Urs Academy of Higher Education and Research, Kolar, IND

**Keywords:** chronic acl injury, medial meniscus injury, lateral meniscus injury, arthroscopy, acl injury

## Abstract

Introduction: In today's orthopaedic practice, meniscus and anterior cruciate ligament (ACL) injuries are prevalent. It is more common in sports injuries and automobile accidents. In patients with ACL tears, meniscus problems are also common. Several studies have linked meniscus injuries to the development of osteoarthritis in early stages. Early onset of osteoarthritis has been observed in ACL-repaired patients with meniscus tears. As a result, meniscus tears should be detected and corrected as soon as possible to avoid degenerative changes in the knee joint. The goal of the study was to see if there was a link between meniscus injuries and ACL injuries in our rural community.

Methods: This retrospective study was conducted on 48 patients at the R.L. Jalappa Hospital & Research Centre from January 2012 to December 2019. Patients between the ages of 18-65 diagnosed with ACL tear with/without meniscus damage in their knees were included in the study. Patients with posterior cruciate ligament (PCL) tear, medial collateral ligament (MCL) or lateral collateral ligament (LCL) injury, previously operated index knees, and patients with more than grade 3 knee osteoarthritis (Kellgren and Lawrence classification) were excluded. Clinical assessment of patients with knee injuries, MRI and diagnostic arthroscopy of the knee joint details were collected. After obtaining the data, we estimated the incidence of meniscus injuries associated with chronic ACL injuries. We also calculated the percentage of side/laterality injury (medial meniscus or lateral meniscus) and part of meniscus injury (anterior horn, body, and posterior horn) in chronic ACL-deficient knees. Patient characteristics such as age and gender were correlated with functional knee assessment using a modified Lysholm score. P-value and chi-square tests were used to assess the data. P-value of less than 0.5 was considered significant.

Results: Average age of the participants was 32.31 years. There were 42 men and six women in the group. Isolated medial meniscus, lateral meniscus, and combined medial and lateral meniscus had average ages of 31.83, 31.16, and 40.28 years, respectively. ACL injuries on the right side were seen in 23 patients the left side was seen in 25 patients. In comparison to the right side, the isolated medial meniscus injury on the left side was more severe. In case of a combination of medial and lateral meniscus tears, the right side suffered more damage than the left. Eleven patients had an isolated ACL tear without a meniscus injury, while the other 37 had a meniscus injury and an ACL tear. Excellent scores were observed in 11 cases, satisfactory scores in 26, and unsatisfactory in 11 cases. Age group was categorized into three groups, less than 30, 30-45, and more than 45 years. Excellent, satisfactory, and unsatisfactory scores were observed in seven, eleven, and seven patients on the left side and excellent, satisfactory, and unsatisfactory scores in four, fifteen, and four patients on the right side, respectively. P-value was not statistically significant when comparing outcomes by age, gender, or side.

Conclusion: Meniscus injuries occurred 77% of the time when there was a persistent ACL injury. In comparison to a lateral meniscus injury, the incidence of medial meniscus injury associated with chronic ACL tear was higher. In comparison to the anterior horn and body of the medial meniscus, the majority of medial meniscus tears were found in the posterior horn.

## Introduction

The anterior cruciate ligament (ACL) is the primary stabilizer of the knee joint; the meniscus is the secondary stabilizer in the anterior-posterior direction. The meniscus becomes the main stabilizer of the knee joint when there is chronic ACL tear or loss of ACL function [1,2]. Damage to both of these structures might compromise knee joint stability and affects joint mobility [3,4]. Meniscal injuries are seen more commonly in contact sports injuries and road traffic accidents [5]. Meniscus injuries are concomitantly associated with ACL injuries [6]. Diagnosis of meniscal injuries or internal derangement of the knee can be done by MRI or diagnostic arthroscopy knee. MRI knee is non-invasive and can be used as a screening tool before therapeutic intervention; MRI is more accurate in diagnosing meniscus and ACL injuries preoperative [7]. MRI is an important screening tool than therapeutic arthroscopy [7]. In some studies, it has been observed that meniscus injuries are associated with the early development of osteoarthritis. In ACL-reconstructed patients with meniscus injuries, poor outcomes with early onset of osteoarthritis are observed [8,9]. Hence, nowadays, meniscal tissue is being preserved as much as possible, and meniscus repair is preferred to meniscectomy [10,11]. The complexity of meniscus tears increases in chronic ACL tears; it becomes very difficult to repair the meniscus in chronic cases [12]. Hence, a meniscus tear should be identified and repaired early to prevent degenerative changes in the knee joint. The purpose of the study was to evaluate the incidence of meniscus injuries associated with chronic ACL injuries, to calculate the percentage of side/laterality injury (medial meniscus or lateral meniscus) and part of meniscus injury (anterior horn, body, and posterior horn) in chronic ACL-deficient knees among our rural population.

## Materials and methods

This retrospective investigation was conducted on 48 patients at R.L. Jalappa Hospital & Research Centre, Kolar, from January 2012 to December 2019. Ethical clearance for our study was obtained from the institutional ethical committee at Sri Devaraj Urs Medical College, Tamaka, Kolar with approval number SDUMC/KLR/IEC/92/2020-21. Patients between the ages of 18 and 65 diagnosed with ACL tear with or without meniscus damage in their knees were included in the study. Patients with posterior cruciate ligament (PCL) tear, medial collateral ligament (MCL) or lateral collateral ligament (LCL) injury, previously operated index knees, and patients with more than grade 3 knee osteoarthritis (Kellgren and Lawrence classification) were excluded. Clinical assessment of patients with knee injuries, MRI of the knee joint and diagnostic arthroscopy of the knee joint details were collected. After obtaining the required data, we estimated the incidence of meniscus injuries associated with chronic ACL injuries. We also calculated the percentage of side/laterality injury (medial meniscus or lateral meniscus) and part of meniscus injury (anterior horn, body, and posterior horn) in chronic ACL-deficient knees. Further patient characteristics such as age and gender were correlated with functional knee assessment using a modified Lysholm score. In the modified Lysholm score, the maximum score is 100 points, between 91-100 is considered excellent, 65-90 as satisfactory, and less than 64 as unsatisfactory. Based on the score, patients were categorized into excellent, satisfactory, and unsatisfactory. P-value and chi-square tests were used to assess the data. P-value of less than 0.5 was considered significant.

## Results

Our study population included 48 patients with chronic ACL tears. Patients with more than six-week-old injuries were considered chronic ACL tears. Out of 48 patients, 42 were males and six were females. In isolated medial meniscus injury, there were 24 patients out of which 22 were males and two were females. In isolated lateral meniscus injury, five were males and one female. In combined medial and lateral meniscus injury, six were males and one female. Among all groups, there was a male preponderance.

The average age group of the study population was 32.31 years. The average age group of isolated medial meniscus, lateral meniscus, and combined medial and lateral meniscus were 31.83 years, 31.16 years, and 40.28 years respectively. Gender preponderance and functional knee assessment based on modified Lysholm score are shown in Table 1. Mode of injuries was classified into sports and non-sports injuries. Sports injuries including contact and non-contact sports were observed in 17 patients which accounted for 35.41% and non-sports injuries which included road traffic accident, fall from a two-wheeler, fall from height, and fall following slipping or tripping was seen in 31 patients which accounted for 64.59%. In isolated medial meniscus injury, eight patients sustained injuries due to sports activity which accounted for 33.33%, and 16 patients sustained injuries due to non-sports activities which accounted for 66.67%. In isolated lateral meniscus injuries, sports activity accounted for 33.33% and non-sports activity accounted for 66.67%. In combined medial and lateral meniscus injury, sports activity accounted for 28.57% and non-sports activity accounted for 71.43%. The distribution of meniscal tears based on laterality is shown in Table 2.

**Table 1:** Gender preponderance and functional knee assessment based on modified Lysholm score [13] The p-value for gender in Pearson chi-square and likelihood ratio were 0.806 and 0.802, respectively, and were not statistically significant.

		GENDER		Total
		F	M	
OUTCOMES	EXCELLENT	1	10	11
	SATISFACTORY	4	22	26
	UNSATISFACTORY	1	10	11
Total		6	42	48

**Table 2:** Distribution of meniscal tear based on laterality

Overall chronic ACL injured patients	48
Isolated medial meniscus tear	24
Isolated lateral meniscus tear	6
Combined medial and lateral meniscus tear	7
Isolated ACL tear	11

**Figure 1:**
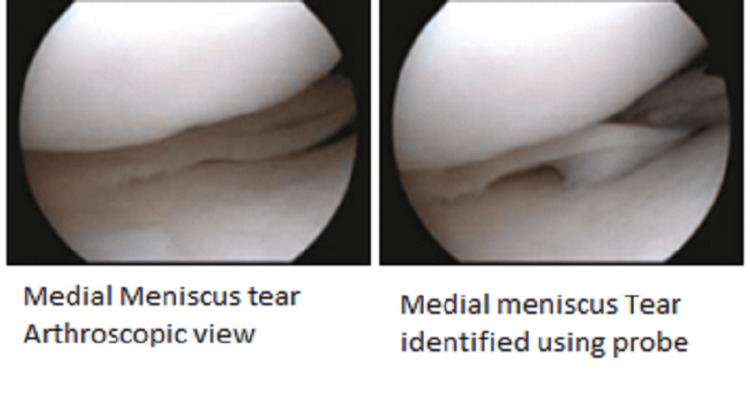
Arthroscopic view of medial meniscus tear

Out of 48 patients, 11 patients had isolated ACL tears with no meniscus injury and the other 37 patients had meniscus injury with concomitant ACL tears. A total of 44 meniscal tears were observed in 37 patients who sustained concomitant chronic ACL tears. Overall medial meniscus injury was 31 out of 44 meniscal injuries and lateral meniscus injury was 13 out of 44 meniscal injuries. Arthroscopic view of the medial meniscus tear is shown in Figure 1.

Out of 37 patients who sustained meniscal tear, 24 patients had isolated medial meniscus tears, six patients had a lateral meniscus tear and seven patients had both medial and lateral meniscus tears. The percentage of meniscal tears observed in the study population was 77.1% (37/48). Incidence of isolated medial meniscal tear was 50% (24/48), isolated lateral meniscal tear was 12.5% (6/48), and combined medial and lateral meniscal tear was 14.6% (7/48). Incidence of overall medial meniscus injury was 70.45% and lateral meniscus injury was 29.55%.

**Figure 2:**
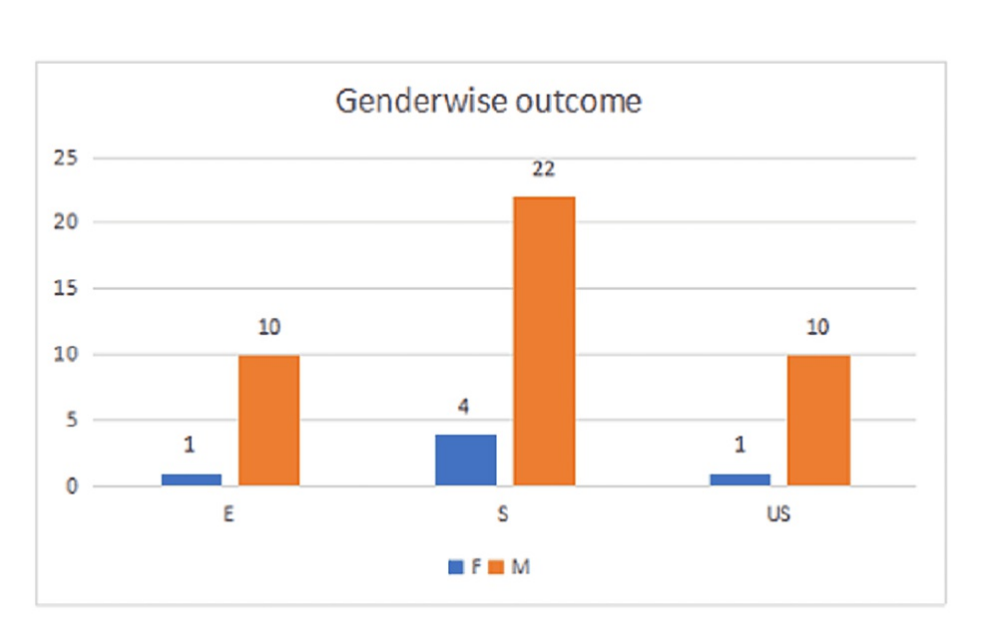
Bar diagram depicting the gender-wise outcomes in association of meniscus injuries in anterior cruciate ligament-injured patients E - Excellent; S - Satisfactory; US - Unsatisfactory; F - Female; M - Male.

Based on the location of the meniscus tear, posterior horn tear of medial meniscus was seen in 22 out of 31 medial meniscus injuries which accounted for 71%, whereas, anterior horn and body tear of medial meniscus was seen in nine which accounted for 29% (Table 3). Lateral meniscus posterior horn tear was seen in eight out of 13 lateral meniscus injuries which accounted for 61.5% (8/13) whereas, anterior horn and body tear of lateral meniscus was seen in five which accounted for 38.5%. The gender-wise outcomes in the association of meniscus injuries in ACL injured patients are shown in Figure 2.

**Table 3:** Injuries based on the location of meniscus tear

Part of Meniscus	Isolated Medial Meniscus tear + Combined Meniscus tear	Isolated Lateral Meniscus + Combined Meniscus tear
Anterior Horn	2+1	1+2
Body	5+1	1+1
Posterior Horn	17+5	4+4

Based on the modified Lysholm score, a functional knee assessment was performed on our patient. Excellent scores were seen in 11 cases, satisfactory in six cases, and unsatisfactory in 11 cases. Age group was categorized into into groups, less than 30 years, 30-45 years, and more than 45 years; scores were assessed in different age groups. Based on the side (right/left) involvement, excellent, satisfactory, and unsatisfactory scores were observed in seven, eleven, and seven patients on the left side, and excellent, satisfactory, and unsatisfactory scores in four, fifteen, and four patients on the right side, respectively. In our study, the P-value was not statistically significant when comparing scores by age, gender, or side involvement. Every attempt was made intra-operatively to conserve the meniscus and repair it. In our study group, medial meniscus was repairable in one case out of 31 medial meniscus injuries which accounted for 3% and lateral meniscus was repairable in one case out of 13 which accounted for 07.6%. In the remaining cases, meniscus was irreparable due to chronic duration of injury and degenerative meniscus. In such cases, the meniscus was debrided and meniscal balancing was done.

## Discussion

We were able to identify the incidence of meniscal injury associated with chronic ACL tears in our rural population. In addition to this, we divided meniscal injuries based on demographic details, side and site affected, and also based on mechanism of injury. In the review of literature, the concomitant meniscal injuries associated with chronic ACL tears occurred in up to 83% [14]. In our study, the incidence of meniscal injuries associated with chronic ACL tears was 77%. The study results by Kilcoyne et al. [14] showed that 56% of tears occurred in the medial meniscus and 44% of tears in the lateral meniscus concomitant with ACL injuries. They also reported that the incidence of medial meniscus injury and lateral meniscus injury was the same which was 56% and 44%, respectively in both acute and chronically injured ACL patients.

Our study results showed that medial meniscus injury accounted for 70.5% (31/44) of total meniscus injured and lateral meniscus injury accounted for 29.5% (13/44). The lateral meniscus is more mobile compared to the medial meniscus and the medial meniscus is adherent to the tibia and is less mobile. Due to this, the medial meniscus is more prone to injury during twisting movements of the knee joint [15]. The study by Thompson et al. showed that in ACL deficient knees, the meniscus has been shown to provide knee stability in anterior-posterior (AP), varus-valgus, and rotational stability in vitro. In chronic ACL deficient knee, the meniscus is under more stress whenever joint movement occurs, especially during twisting movement; therefore, it is more prone to injury [16]. Our study showed that the incidence of medial meniscus injury associated with chronic ACL injury was more compared to a lateral meniscus injury. When there is loss of ACL function/ACL tear, medial meniscus will be the primary stabilizer of the knee joint, mainly in the anterior-posterior direction. The posterior horn of the medial meniscus acts as a wedge or mechanical block between the femur and the tibia when there is tibial translation movement. During this movement, the posterior horn of the medial meniscus is under stress which might result in a posterior horn tear. In our study, we found that 71% (22/31) of medial meniscal tears occurred in the posterior horn of the medial meniscus. The limitation of our study was our small sample size; in our rural population, affordability for MRI investigation and arthroscopy knee is less compared to urban areas. To know the actual incidence of meniscus tears concomitant with ACL tears, a larger population is required.

## Conclusions

Meniscus injuries occurred 77% of the time in our study sample when there was a persistent ACL injury. In comparison to a lateral meniscus injury, the incidence of medial meniscus injury associated with chronic ACL tear was higher. In comparison to the anterior horn and body of the medial meniscus, the majority of medial meniscus tears were found in the posterior horn.
